# Executive Cognitive Functioning and Cardiovascular Autonomic Regulation in a Population-Based Sample of Working Adults

**DOI:** 10.3389/fpsyg.2016.01536

**Published:** 2016-10-05

**Authors:** Cecilia U. D. Stenfors, Linda M. Hanson, Töres Theorell, Walter S. Osika

**Affiliations:** ^1^Aging Research Center, Department of Neurobiology, Care Science and Society, Karolinska InstituteStockholm, Sweden; ^2^Environmental Neuroscience Lab, Department of Psychology, University of ChicagoChicago, IL, USA; ^3^Stress Research Institute, Stockholm UniversityStockholm, Sweden; ^4^Department of Neurobiology, Care Science and Society, Center for Social Sustainability, Karolinska InstituteStockholm, Sweden; ^5^Department of Clinical Neuroscience, Karolinska InstituteStockholm, Sweden

**Keywords:** executive cognitive function, autonomic regulation, heart rate variability, cardiac repolarization, population based

## Abstract

**Objective:** Executive cognitive functioning is essential in private and working life and is sensitive to stress and aging. Cardiovascular (CV) health factors are related to cognitive decline and dementia, but there is relatively few studies of the role of CV autonomic regulation, a key component in stress responses and risk factor for cardiovascular disease (CVD), and executive processes. An emerging pattern of results from previous studies suggest that different executive processes may be differentially associated with CV autonomic regulation. The aim was thus to study the associations between multiple measures of CV autonomic regulation and measures of different executive cognitive processes.

**Method:** Participants were 119 healthy working adults (79% women), from the Swedish Longitudinal Occupational Survey of Health. Electrocardiogram was sampled for analysis of heart rate variability (HRV) measures, including the Standard Deviation of NN, here heart beats (SDNN), root of the mean squares of successive differences (RMSSD), high frequency (HF) power band from spectral analyses, and QT variability index (QTVI), a measure of myocardial repolarization patterns. Executive cognitive functioning was measured by seven neuropsychological tests. The relationships between CV autonomic regulation measures and executive cognitive measures were tested with bivariate and partial correlational analyses, controlling for demographic variables, and mental health symptoms.

**Results:** Higher SDNN and RMSSD and lower QTVI were significantly associated with better performance on cognitive tests tapping inhibition, updating, shifting, and psychomotor speed. After adjustments for demographic factors however (age being the greatest confounder), only QTVI was clearly associated with these executive tests. No such associations were seen for working memory *capacity*.

**Conclusion:** Poorer CV autonomic regulation in terms of lower SDNN and RMSSD and higher QTVI was associated with poorer executive cognitive functioning in terms of inhibition, shifting, updating, and speed in healthy working adults. Age could largely explain the associations between the executive measures and SDNN and RMSSD, while associations with QTVI remained. QTVI may be a useful measure of autonomic regulation and promising as an early indicator of risk among otherwise healthy adults, compared to traditional HRV measures, as associations between QTVI and executive functioning was not affected by age.

## Introduction

Executive cognitive functioning (including working memory processes, attentional, and cognitive control processes like inhibition, shifting attention, updating, and maintenance) is essential in both private life and working life, not least as the majority of jobs in industrialized countries are now characterized by cognitively demanding tasks for which executive cognitive functioning is essential. At the same time, executive cognitive functioning varies both between individuals and within individuals across time and appears to be particularly sensitive to impairments from stress related physiological processes (Sandström et al., 2005; Arnsten, 2009; Liston et al., 2009; Grossi et al., 2015) and the wear and tear with aging (McEwen et al., 2015). Understanding of the mechanisms that affect executive cognitive functioning is therefore important and imperative in strategies for promoting and maintaining executive cognitive functioning throughout life.

It is plausible that autonomic regulation and CV physiology is an underlying factor affecting executive cognitive functioning among working adults, for example as a result of psychosocial/psychological stress (McEwen, 2007; McEwen et al., 2015). Specifically, with long term stress exposure, autonomic regulation may be altered (Chandola et al., 2010), which in turn has negative consequences for CV health (Thayer et al., 2010; Wulsin et al., 2015), as well as cognitive health (Critchley et al., 2013). Related effects also include lowered resilience to stress—i.e., less adaptive and flexible responding to challenges (Ursin and Eriksen, 2010), stronger or altered emotional (Golkar et al., 2014), and physiological (Golkar et al., 2014; McEwen et al., 2015; Peters and McEwen, 2015) reactions to stressors—which may contribute further to deteriorating CV and brain health.

CV health has been shown to be important for cognitive health (Gardener et al., 2016), and e.g., mid-life vascular risk factors have also been associated with later mild cognitive impairment and dementia (Kivipelto et al., 2001a,b; Gottesman et al., 2014). Similarly, job-stress has been found to be associated with CV risk factors and cardiovascular disease (CVD) on the one hand (Kivimäki et al., 2006, 2012; Jarczok et al., 2013; Fishta and Backé, 2015; Theorell et al., 2016) as well as to cognitive functioning and dementia on the other hand. For example, mid-life job stress has been found to be a significant predictor of cognitive functioning in old age (Andel et al., 2011) and dementia, including vascular dementia (Andel et al., 2012) and Alzheimers Disease (Wang et al., 2012; Sindi et al., 2014).

Furthermore, executive cognitive functioning appears to be particularly implicated in the common mental health problems experienced in the working population such as chronic stress, exhaustion (Sandström et al., 2005; Grossi et al., 2015), and depressive symptoms (Murrough et al., 2011) that in turn have been found to be associated with CV autonomic regulation and CVD (burnout and exhaustion: De Vente et al., 2003; Brotman et al., 2007; Collins and Karasek, 2010; Olsson et al., 2010; Steptoe and Kivimäki, 2012; depression: Ford et al., 1998; Hughes and Stoney, 2000; Chang et al., 2012; Watkins et al., 2013; Seldenrijk et al., 2015).

Heart rate variability (HRV), the variation in consecutive heartbeat intervals, results from the constant interaction between the sympathetic and parasympathetic arms of the autonomic nervous system and is modulated with e.g., changing environmental demands (Brosschot et al., 2010). Alterations in HRV may thus be an indicator of a maladaptive physiological state in which the balance of sympathetic and parasympathetic processes both at the peripheral and central nervous system level are suboptimal for cognitive functioning. HRV has in several studies been used to assess autonomic imbalances, including psychiatric diseases such as depression, bipolar disorder, and schizophrenia (e.g., Thayer et al., 2010; Montaquila et al., 2015; Bassett et al., 2016), and predict later disease (Tsuji et al., 1994; Liao et al., 1997). One measure of HRV is the Standard Deviation of the interval between normal successive heart-beats (SDNN), and the root of mean squares of successive differences in heart-beat-intervals (RMSSD) is also frequently used. Since particularly HF power band HRV, derived from spectral analyses, has been used in previous studies in relation to cognition and has been argued to more closely measure parasympathetic/vagal activity, this measure was also included in the present study.

Another measure of the ratio of sympathetic to parasympathetic tone and also including the myocardial repolarization pattern is QT variability (QTVI; Berger et al., 1997). QT intervals are sampled on surface ECG tracings and described as a measure of cardiac repolarization lability. QT variability index (QTVI) has been most frequently applied to patients with cardiac and renal disease, and has been used in risk stratification of these patients (Gao et al., 2005; Baumert et al., 2011; Magrì et al., 2012; Dobson et al., 2013; Piccirillo et al., 2013). In psychiatric diseases such as panic disorder and depression, the QTVI has also been shown to be increased (Yeragani et al., 2003; Baumert et al., 2008). Thus, such mental health conditions and symptoms should be controlled for when studying HRV in relation to executive cognitive functioning.

While HRV tends to decrease with age (Vandeput et al., 2008; Voss et al., 2015), it has previously been found to be a more useful (i.e., discriminating) measure of autonomic regulation and CV risk in younger adults than older adults since the latter in general have multiple conditions and diseases that can confound the usefulness of HRV and its associations with other health factors (Antelmi et al., 2004; Vandeput et al., 2008; Mueller et al., 2014). In the present study, we handle this by excluding participants with such potentially confounding health conditions.

Findings from studies of the association between autonomic regulation of the CV system and executive cognitive functioning among adults in the working population have been quite complex and inconclusive (Critchley et al., 2013).

Thayer et al. (2009) have suggested an important relationship among cognitive performance, HRV, and prefrontal neural function based on a number of studies from their group showing that individual differences in HRV are related to performance on tasks associated with executive function and prefrontal cortical activity. For example, in a study of 53 healthy males (highly trained military personnel, which might limit the generalizability of the findings), Hansen et al. (2003) found an association between higher HRV (RMSSD, assessed during the 2-back task) and better performance on a numerical 2-back task (better accuracy), tapping updating, and a Continuous Performance Task (better RT and accuracy). Further, in a similar participant sample, Hansen et al. (2004) found that prospective changes/decreases in the high frequency power band (HF) HRV (the only measure reported) were associated with prospective changes/decreases in performance on these same tasks. Kimhy et al. (2013) found a significant association between resting HRV (HF HRV) and a composite measure of executive function. However, the addition of the demographic covariates (age, sex, education) to the model eliminated the significance of this association. In another experiment cardiac vagal recovery after a cognitive challenge was measured, and better cardiac vagal recovery was associated with better attention-switching and response inhibition abilities. This association remained significant after adding the demographic, clinical, and health behavior covariates. All the other components of the global executive composite measure (i.e., category fluency/“verbal ability and speed,” fluid intelligence/reasoning, working memory capacity/backwards digit span, and basic speed of processing without demands on executive processes/counting backwards) were not associated with the vagal recovery score. Hence, the authors suggest that future studies may need to investigate the links of CV autonomic regulation to specific executive cognitive functions. However, an important limitation of the study by Kimhy et al. (2013) was that the CV control and cognition measures were evaluated on separate occasions, in some cases several years apart (Kimhy et al., 2013). In another recent study by Richard Jennings et al. (2015) assessing the associations between HF HRV and different executive tests in a large American sample (*n* = 440), response inhibition (Stroop performance) was the only measure associated with HF HRV in the whole sample, and was robust to adjustments for demographical and health variables. Among Caucasians (*n* = 363) greater HF HRV was related to better performance on backward digit span (*r* = –0.12, *p* < 0.05), Trail Making Test B/shifting (*r* = –0.12, *p* < 0.05), and Stroop Interference (*r* = 0.19, *p* < 0.01).

Luque-Casado et al. (2016) on the other hand found that the association between HRV and different executive functioning measures vs. control tasks was only dependent on a function of time-on-task. The conclusion was that HRV is highly sensitive to overall demands of sustained attention over and above the influence of other cognitive processes.

In a large sample of U.S. adults, Mann et al. (2015) found a significant association between HF HRV (the only HRV measure reported) and a latent cognitive variable (including a WM test/digit backwards, a Go-NoGo task, and category fluency, often used as a semantic memory test) but no association with the latent cognitive variable after adjustments for demographical factors. However, the basic bivariate correlations reported showed that the only cognitive measure that was significantly associated with HF HRV was the Go-NoGo task which tapped the executive processes of inhibition and attentional set-shifting.

Overall, the results from previous studies seem to point toward a pattern of better CV regulation being associated with primarily better cognitive inhibition, shifting, updating (a key component in e.g., n-back tasks), and in some cases speed, while working memory capacity (the ability to maintain multiple items in short-term memory during simultaneous processing of unrelated information) is not associated with HRV measures to the same extent (an exception being the association between HF HRV and backwards digit span in Richard Jennings et al., 2015). However, since previous studies tend to report on different HRV measures that were measured in different ways (during rest, during cognitive activities, and during recovery) in relation to global or specific executive cognitive function measures, the possibility to draw firm conclusions is yet limited. Furthermore, studies of QTVI in healthy adults, in relation to executive functioning are, to the best of our knowledge, as of yet lacking. Thus, the purpose of the current study was to investigate the association between multiple measures of CV autonomic regulation and different executive cognitive processes in a population-based sample of healthy working adults, while adjusting for important demographic factors, as well as chronic stress and depressive symptoms.

Potentially confounding health conditions were excluded in order to investigate the extent to which the HRV and QTVI measures are associated with specific executive cognitive processes and the extent to which age and other confounders explain these associations among healthy adults.

Based on the existing research on HRV, QTVI, and executive cognitive processes, it was hypothesized that higher HRV (in terms of SDNN, RMSSD, and HF HRV) and lower QTVI would be associated with better executive functioning. Based on the pattern of findings from previous studies, one would expect HRV and QTVI to be particularly associated with performance on tasks that tap the cognitive processes of inhibition, shifting, and updating.

## Methods

### Participants

Participants were recruited from the 2010 wave of the Swedish Longitudinal Occupational Survey of Health (SLOSH). SLOSH is a longitudinal study of work life, social situation and health among Swedish employees, which is conducted biennially and is approximately representative of the Swedish work force (see e.g., Leineweber et al., 2013). SLOSH 2010 participants living in the Stockholm and Gothenburg counties and surrounding counties were invited to an in-depth study of cognitive functioning, with testing taking place in Stockholm at the Stress Research Institute, Stockholm University, or in Gothenburg at the Institute for Stress Medicine).

A total of 233 people participated in the lab study of cognitive functioning, and had reported varying levels of subjective cognitive complaints (SCC), including problems with concentration, memory, decision-making, and thinking clearly (four items rated on a 5-point scale regarding frequency during the past 3 months, ranging from “never” to “always”), adopted from Kristensen et al. (2005). Half (117) had reported low levels of SCC (mean rating score ≤ 2.0) and the other half (116) had reported higher levels of SCC (mean rating score ≥3.25), while being similar with regards to age, sex, educational level, and geographical area.

Exclusion criteria were known or probable brain injury, such as prior head trauma, stroke, or chemical poisoning, as well as other significant illness conditions that can have profound impacts on cognition (e.g., psychotic conditions) and are relatively rare in the general working population. Seven individuals were excluded from the study for such reasons.

Out of the remaining 226 participants, 192 had valid ECG recordings from which SDNN, the standard deviation of NN (here R-R intervals) could be extracted/computed. Out of the 192 participants with SDNN data, seven lacked a discernible QT-interval and therefore lacked an estimate of QTVI. Out of these 192 participants, 73 were excluded from the analyses due to reporting the presence of one or more conditions that could affect either or both CV autonomic regulation and executive cognitive performance, including CV diseases/conditions (*n* = 41), psychiatric conditions (*n* = 24), diabetes (*n* = 7), and thyroid disease (hypothyroidism, *n* = 16).

The age range was 25–66 years and 79% were women. For more details on sampling procedure and descriptive statistics for the complete sample, see Stenfors et al. (2013).

### General protocol

Those consenting to participate were given an appointment for in-person-testing at the lab in their geographical area (Stockholm or Gothenburg).

At the test occasion, informed consent was collected by the research nurse. The participant then rested for 2 min in a supine position, after which ECG electrodes were positioned and ECG was recorded for 5 min. Then neuropsychological testing was administered.

### Executive cognitive functioning and speed

The computerized tests were programmed and presented in E-prime 2.0 (Psychology Software Tools Inc., Sharpsburg, PA, USA).

#### The reading span task

The reading span task used was a modified version (Stenfors et al., 2013) of the original test (Daneman and Carpenter, 1980) with recommended modifications (Engle et al., 1999; Conway et al., 2005). This 16-trial test requires the simultaneous maintenance of to-be-remembered items (2, 3, 4, or 5 words per trial) and processing of other information (sentences) and measures verbal working memory (WM) capacity. For each trial, single to-be-remembered words, followed by a nonsense or correct sentence, are presented on a computer screen, and the participant has to respond “Yes” or “No” as to whether each sentence is logically correct or nonsense. When all word-sentence pairs within a trial have been presented the participant has to freely recall the to-be-remembered words in the correct serial order. There were four trials at each load level and the trials at different load levels were mixed throughout the test in order to avoid differential proactive interference for different load levels (Engle et al., 1999; Conway et al., 2005).

The test measures of verbal WM capacity used were (Friedman and Miyake, 2005):

(1) Span level. This is operationalized as the load level (i.e., 2, 3, 4, or 5) at which at least 3 out of 4 trials can be recalled perfectly—i.e., all words within a trial are recalled in the correct order in at least 34 of the trials—and then adding to this number the overall proportion of words recalled correctly at the next load level.
(2) The total number of correctly freely recalled words over all 16 trials.

#### The 2-back task

The 2-back task taps the WM processes of maintenance and updating of verbal stimuli (McElree, 2001; Stenfors et al., 2013).

Forty single words are consecutively shown on a computer screen and the task is to answer for each word whether it is the same as the word shown two items back (22.5% target items/trials) by pressing the “Yes” or the “No” key. The test measures used were (1) mean response accuracy, and (2) mean response time (RT) on the 2-back/target trials. Word stimuli were commonly occurring 1–3 syllable words in Swedish.

#### The trail-making tests (TMT) A and B

The trail-making tests (TMT) A and B assesses psychomotor speed and attentional shifting (Tombaugh, 2003). In TMT A the task is to connect, by making pencil lines, 25 encircled numbers randomly arranged on a page in proper order. Part A gives a baseline measure of perceptual processing and motor speed. TMT B captures attentional shifting as the task is instead to connect 25 encircled numbers and letters in alternating order. The main test measures are the durations to complete the respective test part.

#### Stroop inhibition and stroop-shifting tests

Stroop inhibition and Stroop-shifting tests (Stroop, 1935): In the Stroop tests, single color-words (in incongruent print color) are consecutively presented on a computer screen and the task is to name the color of the print as quickly as possible and override the automatic process of reading the color-word (in the Stroop inhibition test). The Stroop-shifting test is similar but additionally included an attentional set-shifting component as the rules of responding shifted between naming the color of the print and reading the color-word. When the rule of responding was to read out the color-word, this was indicated on the stimulus word by underscore signs, e.g.,: **_green_**

Stimulus word duration was 2500 ms and inter-stimulus interval was 500 ms in each test part.

The Stroop inhibition test contained 20 trials and the Stroop-shifting test contained 28 trials (50% were repeat trials and 50% were shifting trials).

Verbal responses were recorded by the test administrator and response times were recorded from key press responses given simultaneously as the verbal responses.

For each test part, the main performance measures were mean response times (RT), excluding missed trials/non-responses and the first trial. Accuracy was overall near perfect and therefore not included in further analyses.

#### The letter digit substitution task

The Letter Digit substitution task (Jolles et al., 1995) taps multiple executive processes including updating and maintenance of information in short term memory and shifting (e.g., Knowles et al., 2015), but is also considered a test of executive cognitive tempo. Here, participants are presented with a coding key of nine boxes with letters, where each corresponds to a digit (i.e., W = 1, B = 2). The task is then to fill in as many empty boxes as possible with the correct corresponding digit to letters, during 1 min. The test measure is the number of correctly filled in boxes/numbers.

### Potential confounders

Potential confounders that are known to play a role in both cognitive and CV status that were utilized in the study were sex, age, educational level (1 = “up to secondary school/12 years of school,” to 3 = “2 years or more of university level studies”), yearly income (in thousands of Swedish crowns), and self-reported depressive symptoms, chronic stress/burnout symptoms and level of physical activity (1/“Never exercise” to 4/“Exercise regularly”; Takada et al., 2010).

Depressive symptoms were measured by the Major depression inventory (MDI), measuring the degree of depressive symptoms during the past 2 weeks with 10 items rated on a scale of 0–5 (Olsen et al., 2003). The sum of item scores (0–50) was used and indicates the degree of depressive symptoms as none (0–23), mild (20–24), moderate (25–29), or severe (≥30).

Chronic stress in terms of burnout symptoms were measured by the Shirom Melamed Burnout Questionnaire (SMBQ), including 22 items rated on a scale of 1–7. The mean scores were used as indicators of chronic stress experienced during the past month (Grossi et al., 2005; Toker et al., 2005).

SCC at the time of the cognitive testing and ECG measurement was measured by the 6-item cognitive subscale (using the mean score) in SMBQ, assessing difficulties in the past month with concentration, thinking clearly and feeling mentally fatigued.

### Cardiovascular autonomic regulation—ECG measurements

On arrival at the laboratory, subjects rested in the supine position on a stationary bench in a quiet room with a comfortable temperature, for about 2 min. Electrocardiogram measurements for analysis of CV autonomic regulation were thereafter assessed in a supine position during 5 min. Three ECG-electrodes were placed over the left fifth interspace, the right fifth interspace, and over the manubrium, respectively. The ECG-signal was inspected for consistency and level of noise, and the electrodes were adjusted or replaced if the signal turned out to be noisy or abrupt. Participants were informed that their heart activity would be recorded for 5 min, and were instructed not to talk or move excessively, but to relax as much as possible. They were also provided with ear protectors to reduce potential noise. Participants were in a supine position for 5 min prior to the start of the recording.

The ECG-registrations were recorded at a sampling frequency of 1000 Hz and stored on a computer. Each 300 s ECG-recording was inspected for ectopic beats and artifacts, as well as for the correct identification of each R-peak by the software, and non-sinus beats and other artifacts were corrected by interpolation (Birkett et al., 1991; Gao et al., 2005). After preparation of the data, time domain measures of HRV such as SDNN and RMSSD were computed [as well as including measures that were not the primary focus of this study, i.e., power spectral analysis of the frequency domain, that partitions the total variance (the “power”) of a continuous series of beats into its frequency components: Low Frequency (LF), 0.04–0.15 Hz, High Frequency (HF) 0.15–0.4 Hz, and the ratio of LF to HF (LF/HF; Akselrod et al., 1981; Appel et al., 1989)]. A QT interval was identified, a QT wave template was provided to the program, and the QTVI was calculated as the logarithm of the ratio of normalized QT variance to heart rate variance, as described by Berger et al. (1997).

### SDNN definition

SDNN is the standard deviation of NN (here R-R intervals). SDNN reflects all the cyclic components responsible for variability in the period of recording (here 5 min), and represents total variability.

### RMSSD definition

RMSSD is the “root mean square of successive differences,” i.e., the square root of the mean of the squares of the successive differences between adjacent NNs (here R-R intervals).

### QTVI algorithm

First, the time of the “R” wave was obtained using a peak-detection algorithm after which the operator provided the program with the beginning and the end of the QT wave template. This algorithm finds the QT interval for each beat using the time-stretch model. If the operator chooses a longer QT template, all QT intervals will be biased accordingly, but the QT variability measures will be relatively unaffected as we correct the variability for the mean QT interval.

RR interval mean (RRm) and variance (RRv) and QT interval mean (QTm) and variance (QTv) were calculated from the instantaneous HR and QT time series sampled at 4 Hz. A normalized QTVI was then calculated (Berger et al., 1997).

$$\text{QTVI}=\log_{\text{10}}\left[\left(\text{QTv}/{\text{QTm}}^{2}\right)/\left(\text{RRv}/{\text{RRm}}^{2}\right)\right]$$

This index represents the log-ratio between the QT interval and the HR variabilities, each normalized for the corresponding mean. The software used for peak detection and temporal QT interval variability analysis was developed in-house (Birkett et al., 1991; Gao et al., 2005).

### Statistical analyses

Analyses were performed using SPSS 22 software.

Individual ECGs, and values on the QTVI and HRV measures were examined by a medical doctor specialized in cardiology. No participants were judged to have clinically abnormal values. Thus all were included in further standard data screening procedures.

One univariate outlier (±3.29 *SD*), disconnected from the distribution, was found among women in QTVI and was excluded from further analyses.

To test the extent to which QTVI and HRV (in terms of SDNN, RMSSD, and HF HRV) were associated with executive cognitive functioning in the hypothesized direction, bivariate correlations were calculated (with 1-tailed significance testing, since directionality of associations was hypothesized a priori). Correlations between the demographic and health control variables (potential confounders) with the cognitive measures, QTVI, SDNN, RMSSD, and HF HRV were also calculated to evaluate the extent to which these were confounders in the associations between the CV autonomic regulation measures and cognitive functioning measures. Where a significant association was found between a HRV measure and cognitive measures, partial correlations were calculated with adjustments being made for relevant confounders (i.e., those control variables that were bivariately correlated with both some CV autonomic regulation measures and cognitive measures, at an alpha level ≤ 0.05).

To control for multiple testing, Benjamini and Hochberg's (1995)False Detection Rate (FDR) procedure was applied to each QTVI/HRV measure separately, to calculate a corrected threshold *p*-value at which overall alpha for the multiple partial correlations with each QTVI/HRV measure is kept at 0.05. The partial correlations that are statistically significant at the 0.05 level after FDR correction are starred in **Table 3**.

### Ethics statement

The study has been approved by the Regional Research Ethics Board in Stockholm (Dnr 2010/397-31).

All study participants have given their written informed consent, in accordance with the Declaration of Helsinki. Data were analyzed anonymously.

## Results

Descriptive statistics for the study measures are shown in Table 1, bivariate correlations between the study measures are shown in Table 2 and partial correlations with adjustment for confounders are shown in Table 3.

**Table 1:** **Descriptive statistics for the study sample**.

**Measure**	***N***	**Prevalence (%)**	**Mean**	**Std. dev**.	**Min**.	**Max**.
SDNN	119	–	36.88	12.99	12.99	79.12
QTVI	114	–	−1.04	0.39	−1.82	0.11
RMSSD	119	–	24.27	11.64	3.11	66.67
High frequency HRV	118	–	338.01	449.21	2.44	2890.00
Letter digit, total correct	118	–	37.00	5.67	25.00	53.00
Reading span, Span score	115	–	2.61	0.64	1.00	4.50
Reading span, total FRC	116	–	35.79	6.47	22.00	50.00
Stroop incongruent, RT (ms)	113	–	737.60	208.79	248.00	1429.00
Stroop shifting, RT (ms)	114	–	991.63	235.39	408.00	1625.00
2-Back2, RT (ms)	117	–	1105.16	230.52	543.11	1603.11
2-Back, accuracy (% correct)	117	–	0.84	0.16	0.33	1.00
TMT A (s)	119	–	26.43	8.35	14.00	51.00
TMT B (s)	119	–	58.83	22.96	25.00	148.00
TMT B-A (s)	119		32.40	21.06	4.00	117.00
MDI rating, sum (0–50)	119	–	8.55	8.10	0.00	32.00
SMBQ, mean score (1–7)	119	–	3.11	1.47	1.00	6.45
SCC, mean score (1–7)	118	–	2.94	1.61	1.00	7.00
Age	119	–	47.98	10.49	25.00	66.00
Income (1000's of SEK/year)	119	–	352.24	166.15	32.00	1226.00
Systolic blood pressure	116	–	119.57	14.12	91.00	155.00
Diastolic blood pressure	116	–	76.84	8.75	60.00	100.00
Physical activity	119	–	3.38	0.77	1.00	4.00
Sex	119	–	–	–	–	–
Women	94	79	–	–	–	–
Men	25	21	–	–	–	–
Educational level	119	–	–	–	–	–
Up to 12 years of school	41	34.5	–	–	–	–
Undergrad. ≤ 2 years	13	10.9	–	–	–	–
Undergrad. >2 years	65	54.6	–	–	–	–

*SDNN, standard deviation of the inter-heart-beat interval; QTVI, cardiac repolarization variability index; RMSSD, root of the mean squares of inter-heart-beat intervals; Total FRC, number of words in the test that were Freely Recalled Correctly; MDI, major depression inventory; SMBQ, Shirom Melamed Burnout Questionnaire; BP, blood pressure*.

**Table 2:** **Bivariate correlations between study variables**.

**Variable**		**SDNN**	**QT VI**	**RMSSD**	**HF HRV**	**Letter digit**	**RS, span score**	**RS, FRC**	**Stroop inhibition, RT**	**Stroop shifting, RT**	**2-Back, RT**	**2-Back, Accuracy**	**TMT A (s)**	**TMT B (s)**	**TMT B–TMT A (s)**	**MDI**	**SMBQ**	**Age**	**Income**	**Phys. activity**	**Sex**	**Edu. level**
SDNN	Pearson's *r*	1																				
	Sig. (1-tailed)																					
	*N*	119																				
QT VI	Pearson's *r*	−0.427^**^	1																			
	Sig. (1-tailed)	0.000																				
	*N*	114	114																			
RMSSD	Pearson's *r*	0.787^**^	−0.426^**^	1																		
	Sig. (1-tailed)	0.000	0.000																			
	*N*	119	114	119																		
HF HRV	Pearson's *r*	0.478^**^	−0.291^**^	0.649^**^	1																	
	Sig. (1-tailed)	0.000	0.001	0.000																		
	*N*	118	114	118	118																	
Letter digit	Pearson's *r*	0.191^*^	−0.177^*^	0.177^*^	0.013	1																
	Sig. (1-tailed)	**0.019**	**0.031**	**0.027**	0.444																	
	*N*	118	113	118	117	118																
Reading span, span score	Pearson's *r*	−0.021	−0.059	0.027	−0.010	0.261^**^	1															
	Sig. (1-tailed)	0.412	0.271	0.389	0.456	0.002																
	*N*	115	110	115	114	115	115															
Reading span, FRC	Pearson's *r*	−0.002	−0.067	0.067	−0.011	0.392^**^	0.717^**^	1														
	Sig. (1-tailed)	0.492	0.243	0.239	0.455	0.000	0.000															
	*N*	116	111	116	115	116	115	116														
Stroop inhibition, RT	Pearson's *r*	−0.225^**^	0.287^**^	−0.247^**^	−0.111	−0.541^**^	−0.131	−0.192^*^	1													
	Sig. (1-tailed)	**0.008**	**0.001**	**0.004**	0.123	0.000	0.086	0.021														
	*N*	113	108	113	112	113	111	112	113													
Stroop shifting, RT	Pearson's *r*	−0.184^*^	0.264^**^	−0.222^**^	−0.085	−0.485^**^	−0.193^*^	−0.320^**^	0.785^**^	1												
	Sig. (1-tailed)	**0.025**	**0.003**	**0.009**	0.186	0.000	0.021	0.000	0.000													
	*N*	114	109	114	113	114	112	113	113	114												
2-Back, RT	Pearson's *r*	−0.134	0.228^**^	−0.076	0.040	−0.442^**^	−0.197^*^	−0.208^*^	0.326^**^	0.400^**^	1											
	Sig. (1-tailed)	0.075	**0.008**	0.206	0.336	0.000	0.018	0.013	0.000	0.000												
	*N*	117	112	117	116	117	114	115	112	113	117											
2-Back, Accuracy	Pearson's *r*	0.062	−0.198^*^	0.119	−0.014	0.297^**^	0.173^*^	0.202^*^	−0.232^**^	−0.227^**^	−0.386^**^	1										
	Sig. (1-tailed)	0.254	**0.018**	0.101	0.442	0.001	0.033	0.015	0.007	0.008	0.000											
	N	117	112	117	116	117	114	115	112	113	117	117										
TMT A (s)	Pearson's *r*	−0.185^*^	0.238^**^	−0.156^*^	−0.115	−0.621^**^	−0.273^**^	−0.286^**^	0.385^**^	0.414^**^	0.390^**^	−0.258^**^	1									
	Sig. (1-tailed)	**0.022**	**0.005**	**0.045**	0.108	0.000	0.002	0.001	0.000	0.000	0.000	0.002										
	*N*	119	114	119	118	118	115	116	113	114	117	117	119									
TMT B (s)	Pearson's *r*	−0.275^**^	0.183^*^	−0.246^**^	0.035	−0.486^**^	−0.285^**^	−0.378^**^	0.362^**^	0.391^**^	0.339^**^	−0.365^**^	0.401^**^	1								
	Sig. (1-tailed)	**0.001**	**0.026**	**0.003**	0.352	0.000	0.001	0.000	0.000	0.000	0.000	0.000	0.000									
	*N*	119	114	119	118	118	115	116	113	114	117	117	119	119								
TMT B–TMT A (s)	Pearson's *r*	−0.227^**^	0.108	−0.206^*^	0.084	−0.282^**^	−0.197^*^	−0.293^**^	0.228^**^	0.248^**^	0.215^**^	−0.296^**^	0.040	0.932^**^	1							
	Sig. (1-tailed)	**0.007**	0.127	**0.012**	0.183	0.001	0.018	0.001	0.008	0.004	0.010	0.001	0.332	0.000								
	*N*	119	114	119	118	118	115	116	113	114	117	117	119	119	119							
MDI, rating	Pearson's *r*	0.128	−0.070	0.143	0.324^**^	−0.065	−0.053	−0.177^*^	0.029	0.104	0.031	−0.042	−0.096	0.035	0.076	1						
	Sig. (1-tailed)	0.083	0.230	0.060	0.000	0.243	0.288	0.029	0.379	0.135	0.369	0.327	0.149	0.353	0.205							
	N	119	114	119	118	118	115	116	113	114	117	117	119	119	119	119						
SMBQ	Pearson's *r*	0.084	−0.014	0.081	0.276^**^	−0.121	−0.102	−0.180^*^	0.051	0.108	0.049	−0.031	−0.105	0.097	0.147	0.845^**^	1					
	Sig. (1-tailed)	0.183	0.443	0.190	0.001	0.095	0.140	0.026	0.294	0.127	0.300	0.372	0.128	0.148	0.055	0.000						
	*N*	119	114	119	118	118	115	116	113	114	117	117	119	119	119	119	119					
Age	Pearson's *r*	−0.315^**^	0.193^*^	−0.424^**^	−0.455^**^	−0.296^**^	−0.174^*^	−0.182^*^	0.355^**^	0.417^**^	0.294^**^	−0.168^*^	0.409^**^	0.327^**^	0.194^*^	−0.230^**^	−0.186^*^	1				
	Sig. (1-tailed)	0.000	0.020	0.000	0.000	0.001	0.031	0.025	0.000	0.000	0.001	0.035	0.000	0.000	0.017	0.006	0.022					
	*N*	119	114	119	118	118	115	116	113	114	117	117	119	119	119	119	119	119				
Income	Pearson's *r*	−0.090	0.036	−0.156^*^	−0.148	−0.007	0.083	0.086	0.091	0.100	−0.042	−0.056	0.056	0.004	−0.017	−0.140	−0.152^*^	0.282^**^	1			
	Sig. (1-tailed)	0.164	0.351	0.045	0.055	0.468	0.188	0.178	0.168	0.145	0.325	0.276	0.271	0.481	0.425	0.064	0.049	0.001				
	*N*	119	114	119	118	118	115	116	113	114	117	117	119	119	119	119	119	119	119			
Physical activity	Pearson's *r*	0.054	−0.069	0.075	0.014	0.006	−0.060	−0.045	−0.062	0.020	−0.109	0.065	0.135	−0.103	−0.166^*^	0.037	−0.052	0.139	0.073	1		
	Sig. (1-tailed)	0.279	0.233	0.210	0.441	0.475	0.262	0.316	0.256	0.416	0.121	0.243	0.071	0.132	0.035	0.344	0.286	0.065	0.215			
	*N*	119	114	119	118	118	115	116	113	114	117	117	119	119	119	119	119	119	119	119		
Sex	Spearman's rho	0.179^*^	−0.114	0.228^**^	0.253^**^	0.215^**^	0.022	0.116	−0.244^**^	−0.245^**^	−0.053	0.212^*^	−0.004	−0.173^*^	−0.212^*^	0.029	0.020	−0.159^*^	−0.219^**^	0.094	1	
	Sig. (1-tailed)	0.026	0.114	0.006	0.003	0.010	0.407	0.108	0.005	0.004	0.283	0.011	0.484	0.030	0.010	0.378	0.414	0.042	0.008	0.154		
	*N*	119	114	119	118	118	115	116	113	114	117	117	119	119	119	119	119	119	119	119	119	
Educational level	Spearman's rho	0.112	−0.077	0.025	−0.018	0.080	0.054	0.104	−0.149	−0.076	−0.037	0.011	0.046	−0.086	−0.126	−0.085	−0.132	0.009	0.206^*^	0.184^*^	0.059	1
	Sig. (1-tailed)	0.113	0.207	0.394	0.425	0.196	0.282	0.133	0.057	0.211	0.347	0.453	0.310	0.176	0.086	0.180	0.076	0.460	0.012	0.022	0.263	
	*N*	119	114	119	118	118	115	116	113	114	117	117	119	119	119	119	119	119	119	119	119	119
SCC	Pearson's *r*	0.132	−0.013	0.110	0.291^**^	−0.163	−0.137	−0.226^*^	0.102	0.175	0.092	−0.082	−0.067	0.163	0.204^*^	0.813^**^	0.912^**^	−0.163	−0.16	−0.035	−0.034	−0.144
	Sig. (1-tailed)	0.155	0.889	0.234	0.001	0.079	0.146	0.015	0.286	0.064	0.326	0.381	0.473	0.078	0.026	0.000	0.000	0.08	0.084	0.705	0.357	0.06
	*N*	118	113	118	117	117	114	115	112	113	116	116	118	118	118	118	118	118	118	118	118	118

** *Correlation is significant at the 0.01 level (1-tailed)*.

* *Correlation is significant at the 0.05 level (1-tailed)*.

*Significant correlations between cardiovascular autonomic regulation measures and cognitive measures are printed in bold*.

*SDNN, standard deviation of the inter-heart-beat interval; QT VI, cardiac repolarization variability index; RMSSD, root of the mean squares of inter-heart-beat intervals; HF HRV, high frequency band heart rate variability; RS, Reading span; Total FRC, number of words in the test that were Freely Recalled Correctly; MDI, major depression inventory; SMBQ, Shirom Melamed Burnout Questionnaire; SCC, subjective cognitive complaints*.

**Table 3:** **Partial correlations between cognitive measures and HRV measures, adjusting for age and sex (1-tailed sign. testing)**.

**Cognitive measure**		**SDNN**	**RMSSD**	**QTVI**
Letter Digit, total correct	Correlation	0.112	0.045	−0.129
	*p*	0.123	0.322	0.098
	df	106	106	101
Reading span, span score	Correlation	−0.022	0.003	−0.088
	*p*	0.411	0.488	0.189
	df	106	106	101
Reading span, total FRC	Correlation	0.001	0.041	−0.104
	*p*	0.497	0.338	0.147
	df	106	106	101
Stroop inhibition, RT (ms)	Correlation	−0.087	−0.055	0.231
	*p*	0.185	0.286	0.009^*^
	df	106	106	101
Stroop shifting, RT (ms)	Correlation	−0.044	−0.001	0.218
	*p*	0.324	0.498	0.014^*^
	df	106	106	101
2-Back, RT (ms)	Correlation	−0.105	0.025	0.252
	*p*	0.139	0.398	0.005^*^
	df	106	106	101
2-Back, accuracy (% correct)	Correlation	−0.052	0.007	−0.196
	*p*	0.295	0.472	**0.024**
	df	106	106	101
TMT A (s)	Correlation	−0.076	0.028	0.179
	*p*	0.217	0.386	**0.035**
	df	106	106	101
TMT B (s)	Correlation	−0.166	−0.109	0.074
	*p*	**0.043**	0.130	0.229
	df	106	106	101
TMT B-A (s)	Correlation	−0.149	−0.130	0.007
	*p*	0.062	0.089	0.471
	df	106	106	101

*SDNN, standard deviation of the inter-heart-beat interval; QTVI, cardiac repolarization variability index; RMSSD, root of the mean squares of inter-heart-beat intervals; Total FRC, number of words in the test that were Freely Recalled Correctly; RT, response time; TMT A and B, Trail Making Test A and B*.

*Partial correlations between QTVI, HRV measures, and Executive cognitive measures*.

*Bolded p-values indicate that p ≤ 0.05 before adjustment for multiple testing*.

* *indicate that p ≤ 0.05 after FDR adjustment for multiple testing*.

Higher SDNN and RMSSD and lower QTVI were significantly bivariately correlated with better executive cognitive performance on Letter Digit, Stroop inhibition, Stroop Shifting, TMT A and B, while only SDNN and RMSSD were associated with TMT B-A, and only lower QTVI was associated with better performance on 2-Back (both RT and accuracy). No associations were found between the CV autonomic regulation measures and Reading span performance.

HF HRV was not associated with the executive cognitive measures.

As expected, age was negatively associated with SDNN (*r* = −0.315, *p* < 0.001), RMSSD (*r* = −0.424, *p* < 0.001) and HF HRV (*r* = −0.455, *p* < 0.001), and positively associated with QTVI (*r* = 0.192, *p* = 0.02). As such, among the CV autonomic regulation measures, QTVI was the least correlated with age. Age was also correlated with several of the cognitive measures.

Sex was also significantly associated with both CV autonomic regulation measures (SDNN, RMSSD, and HF HRV) and cognitive measures, while educational level and physical activity were not. Income was only associated with RMSSD but not with any of the cognitive measures and thus these variables did not meet the criteria of a confounder.

Therefore, in the analyses of partial correlations between the CV autonomic regulation measures and the cognitive measures, statistical adjustments were made for age and sex.

After adjustment for age and sex, the associations between SDNN and RMSSD with these cognitive measures were no longer significant, except an association between higher SDNN and better performance (less time to complete task) on TMT B (*r* = −0.166, *p* = 0.043). Higher QTVI, on the other hand, was still clearly associated with poorer performance on Stroop inhibition (*r* = 0.231, *p* = 0.009), Stroop shifting (*r* = 0.218, *p* = 0.014), 2-back (longer RT: *r* = 0.252, *p* = 0.005; lower accuracy: *r* = −0.196, *p* = 0.024) and TMT A (slower completion time, *r* = 0.179, *p* = 0.035), even after adjustments for the confounders age and sex. See Table 3 for results on the partial correlations. By definition, the symptoms of depression, chronic stress/burnout, and cognitive complaints were not confounders between SDNN, RMSSD, QTVI, and the cognitive measures, and additionally adjusting for these did not alter the associations between QTVI and the cognitive measures, but only marginally changed the association between SDNN and TMT B such that it was no longer significant.

After applying the FDR procedure to correct for multiple testing, QTVI was still significantly associated with performance on Stroop inhibition, Stroop Shifting, and 2-Back (RT).

## Discussion

The general aim of the present study was to investigate the extent to which CV autonomic regulation, including both traditional measures of HRV as well as cardiac repolarization patterns which has not been studied in healthy populations, is associated with specific executive cognitive processes in a healthy sample of the working population.

In accordance with the a priori predictions, it was found that higher SDNN and RMSSD and lower QTVI were associated with better executive cognitive functioning, specifically better inhibition, shifting, updating and psychomotor speed, while no associations were found with working memory capacity (measured by the Reading span task). HF HRV on the other hand was not associated with the executive cognitive measures.

These findings resemble the pattern of findings in previous studies of HRV and executive functioning, wherein higher HRV (measured by SDNN, RMSSD, or HF HRV during rest, cognitive activity, or during recovery) tends to be associated primarily with performance on executive tasks that tap inhibition, shifting, and updating an sometimes speed, while not being associated with tests of working memory capacity (i.e., how many items can be maintained in short-term memory while simultaneously processing/manipulating other information; Hansen et al., 2003, 2004; Kimhy et al., 2013; Mann et al., 2015; Richard Jennings et al., 2015). Regarding associations between HRV measures and working memory measures, there seems to be a divide between tasks that tap the *capacity* component of working memory (i.e., the maintenance of multiple items in short-term memory in the face of distraction) contra other processes involved in working memory such as updating (on which there is more emphasis in e.g., n-back tasks). The latter rather than the former repeatedly appears in prior research to be associated with HRV (Hansen et al., 2003, 2004; Kimhy et al., 2013; Mann et al., 2015), the finding of an association between HF HRV and backwards digit span being an exception (Richard Jennings et al., 2015).

There are several important determinants of HRV. The most significant one seems to be the age related decline in autonomic function and thus in HRV (Vandeput et al., 2008; Voss et al., 2015). Accordingly, in this study there was a consistent decline in SDNN with increasing age, as well as a clear association with increasing age and higher QTVI, which is in line with earlier studies (Piccirillo et al., 2001; Hasan et al., 2012; Kimhy et al., 2013; Mann et al., 2015). However, among those measures, QTVI had the smallest correlation with age.

Accordingly, the associations between HRV (SDNN and RMSSD) and the executive cognitive measures could largely be accounted for by age. QTVI, however, was clearly associated with performance on tests tapping inhibition (Stroop inhibition), shifting (Stroop shifting), updating and context maintenance (2-back), even after adjustments for demographical factors, as well as symptoms of chronic stress and depression.

QTVI thus seems to be a promising measure of CV autonomic regulation that is less confounded by age, but which still has implications for cognitive health. This might be due to the fact that the QTVI mirrors the cardiac repolarization process, which may be a better predictor of autonomic influence on psychological functioning, compared with other HRV measures used in earlier research (e.g., Kimhy et al., 2013; Mann et al., 2015).

An increase in QTVI has also been shown in anxiety (Yeragani et al., 2003) and depression (Baumert et al., 2008; Koschke et al., 2009), which in turn have been found to be major risk factors for cognitive decline and dementia (Brommelhoff et al., 2009; Gallacher et al., 2009; Petkus et al., 2015). However, in the present study, symptoms of depression and stress/burnout were not associated with QTVI and generally did not affect the associations between HRV, QTVI, and the executive cognitive measures.

To our knowledge, this is the first time that QTVI, in addition to traditional HRV measures of CV autonomic regulation, have been assessed in relation to specific measures of executive function in a population based sample of healthy working adults. Our findings indicate that QTVI may play a role in executive cognitive functioning among working adults, even after adjustments for age, demographical factors, and health variables including depressive and burnout symptoms.

## Limitations

The cross-sectional design does not allow for evaluations of causality and causal directions between SDNN, QTVI, and cognitive variables. It should be emphasized that causality is likely bi-directional. That is, poor executive cognitive functioning could be a factor that contributes to dysregulations in autonomic regulation as well as the other way around. For example, low cognitive ability is associated with increased risks of CVD (Shipley et al., 2008; Batty et al., 2009, 2010), due for example to lower self-control and unhealthy lifestyle behaviors (Batty et al., 2007), as well as potentially higher exposure to psychosocial stressors like poor work-environment and economic resources, which can all have a negative impact on CV health (Batty and Deary, 2004).

The measure of physical exercise was relatively crude and exclusively self-rated and thus the control for this aspect was limited in the present study. The lack of bivariate associations between the physical exercise measure and the measures of autonomic regulation suggests that level of physical activity engagement was not adequately captured. Thus, it is possible that this factor could explain some of the relationship between QTVI and executive cognitive performance in the younger age group.

The Stroop tests required responses to be made verbally and motorically simultaneously. That is, the participant had to name the color of the print out loud, or read the word for some trials in the Stroop Shifting test, while simultaneously pressing the space key, which recorded response times. Although the test administrator was present to ensure that the participant responded in the required manner and that the verbal and key press responses were synchronized, it is possible that there is some error variance in the response times due to this response procedure.

It should also be noted that women were over-represented (79 %) in the participant sample and therefore the results should be interpreted with caution with regards to generalizability among men.

## Conclusion

This study investigated and found that suboptimal CV autonomic regulation, including SDNN, RMSSD, and myocardial repolarization patterns measured by QTVI, are associated with lower performance in specific executive cognitive processes (inhibition, shifting, updating, and speed), in a population based sample of healthy working adults. While age could explain the relationships between SDNN, RMSSD, and the executive cognitive measures, QTVI was clearly associated with the measures of these executive processes even after adjustments for demographical factors, as well as symptoms of depression and chronic stress. QTVI was in this study less dependent on age and may be promising as an early indicator of autonomic regulation that has implications for executive cognitive functioning, relative to other traditional measures of CV autonomic functioning such as HRV (measured by SDNN and RMSSD).

Longitudinal studies that could clarify the prognostic significance of autonomic dysfunction, including QTVI, in terms of adverse cognitive health outcomes are warranted. If clear causal links emerges, interventions that promote better CV autonomic regulation, which in turn might improve executive cognitive functioning or prevent age-related decreases in executive functioning, could become relevant.

## Author contributions

CS conceived and designed the study, collected cognitive and mental health data, performed the data analyses, and wrote the manuscript. WO supervised electrocardiogram data collections and analyses and made important contributions to the study design and manuscript. LH contributed to the interpretation of data analyses and made important intellectual contributions in reviewing the manuscript. TT contributed to the conception of the study, interpretation of data analyses and critical review of the manuscript. All authors reviewed and approved the manuscript for submission.

### Conflict of interest statement

The authors declare that the research was conducted in the absence of any commercial or financial relationships that could be construed as a potential conflict of interest. The reviewer VT and the handling Editor declared their shared affiliation, and the handling Editor states that the process nevertheless met the standards of a fair and objective review.

## References

[B1] Akselrod S. Gordon D. Ubel F. A. Shannon D. C. Barger A. C. Cohen R. J. (1981). Power spectrum analysis of heart rate fluctuations: a quantitative probe of beat-to-beat cardiovascular control. Science 213, 220–222. 10.1126/science.6166045 6166045

[B2] Andel R. Crowe M. Hahn E. A. Mortimer J. A. Pedersen N. L. Fratiglioni L.. (2012). Work-related stress may increase the risk of vascular dementia. J. Am. Geriatr. Soc. 60, 60–67. 10.1111/j.1532-5415.2011.03777.x 22175444 PMC3258308

[B3] Andel R. Crowe M. Kåreholt I. Wastesson J. Parker M. G. (2011). Indicators of job strain at midlife and cognitive functioning in advanced old age. J. Gerontol. Ser. B Psychol. Sci. Soc. Sci. 66, 287–291. 10.1093/geronb/gbq105 21292810

[B4] Antelmi I. De Paula R. S. Shinzato A. R. Peres C. A. Mansur A. J. Grupi C. J. (2004). Influence of age, gender, body mass index, and functional capacity on heart rate variability in a cohort of subjects without heart disease. Am. J. Cardiol. 93, 381–385. 10.1016/j.amjcard.2003.09.065 14759400

[B5] Appel M. L. Berger R. D. Saul J. P. Smith J. M. Cohen R. J. (1989). Beat to beat variability in cardiovascular variables: noise or music? J. Am. Coll. Cardiol. 14, 1139–1148. 10.1016/0735-1097(89)90408-7 2681319

[B6] Arnsten A. F. (2009). Stress signalling pathways that impair prefrontal cortex structure and function. Nat. Rev. Neurosci. 10, 410–422. 10.1038/nrn2648 19455173 PMC2907136

[B7] Bassett D. Bear N. Nutt D. Hood S. Bassett S. Hans D. (2016). Reduced heart rate variability in remitted bipolar disorder and recurrent depression. Aust. N. Z. J. Psychiatry 50, 793–804. 10.1177/0004867416652734 27307288

[B8] Batty G. D. Deary I. J. (2004). Early life intelligence and adult health. BMJ 329, 585–585. 10.1136/bmj.329.7466.585 15361422 PMC516648

[B9] Batty G. D. Deary I. J. Benzeval M. Der G. (2010). Does IQ predict cardiovascular disease mortality as strongly as established risk factors? Comparison of effect estimates using the West of Scotland Twenty-07 cohort study. Eur. J. Cardiovasc. Prev. Rehabil. 17, 24–27. 10.1097/HJR.0b013e328321311b 20101181

[B10] Batty G. D. Deary I. J. Gottfredson L. S. (2007). Premorbid (early life) IQ and later mortality risk: systematic review. Ann. Epidemiol. 17, 278–288. 10.1016/j.annepidem.2006.07.010 17174570

[B11] Batty G. D. Gale C. R. Tynelius P. Deary I. J. Rasmussen F. (2009). IQ in early adulthood, socioeconomic position, and unintentional injury mortality by middle age: a cohort study of more than 1 million Swedish men. Am. J. Epidemiol. 169, 606–615. 10.1093/aje/kwn381 19147741 PMC2640161

[B12] Baumert M. Lambert G. W. Dawood T. Lambert E. A. Esler M. D. McGrane M.. (2008). QT interval variability and cardiac norepinephrine spillover in patients with depression and panic disorder. Am. J. Physiol. Heart Circ. Physiol. 295, H962–H968. 10.1152/ajpheart.00301.2008 18599596

[B13] Baumert M. Schlaich M. P. Nalivaiko E. Lambert E. Sari C. I. Kaye D. M.. (2011). Relation between QT interval variability and cardiac sympathetic activity in hypertension. Am. J. Physiol. Heart Circ. Physiol. 300, H1412–H1417. 10.1152/ajpheart.01184.2010 21257917

[B14] Benjamini Y. Hochberg Y. (1995). Controlling the false discovery rate: a practical and powerful approach to multiple testing. J. R. Stat. Soc. B 57, 289–300.

[B15] Berger R. D. Kasper E. K. Baughman K. L. Marban E. Calkins H. Tomaselli G. F. (1997). Beat-to-beat QT interval variability novel evidence for repolarization lability in ischemic and nonischemic dilated cardiomyopathy. Circulation 96, 1557–1565. 9315547 10.1161/01.cir.96.5.1557

[B16] Birkett C. L. Kienzle M. G. Myers G. A. (1991). Interpolation over ectopic beats increases low frequency power in heart rate variability spectra, in Proceedings of IEEE Computers in Cardiology (Los Alamitos, CA: IEEE Computers in Society Press), 257–259.

[B17] Brommelhoff J. A. Gatz M. Johansson B. McArdle J. J. Fratiglioni L. Pedersen N. L. (2009). Depression as a risk factor or prodromal feature for dementia? Findings in a population-based sample of Swedish twins. Psychol. Aging 24, 373. 10.1037/a0015713 19485655 PMC2713179

[B18] Brosschot J. F. Verkuil B. Thayer J. F. (2010). Conscious and unconscious perseverative cognition: is a large part of prolonged physiological activity due to unconscious stress? J. Psychosom. Res. 69, 407–416. 10.1016/j.jpsychores.2010.02.002 20846542

[B19] Brotman D. J. Golden S. H. Wittstein I. S. (2007). The cardiovascular toll of stress. Lancet 370, 1089–1100. 10.1016/S0140-6736(07)61305-1 17822755

[B20] Chandola T. Heraclides A. Kumari M. (2010). Psychophysiological biomarkers of workplace stressors. Neurosci. Biobehav. Rev. 35, 51–57. 10.1016/j.neubiorev.2009.11.005 19914288 PMC2891393

[B21] Chang H. A. Chang C. C. Chen C. L. Kuo T. B. Lu R. B. Huang S. Y. (2012). Major depression is associated with cardiac autonomic dysregulation. Acta Neuropsychiatr. 24, 318–327. 10.1111/j.1601-5215.2011.00647.x 25287173

[B22] Collins S. Karasek R. (2010). Reduced vagal cardiac control variance in exhausted and high strain job subjects. Int. J. Occup. Med. Environ. Health 23, 267–278. 10.2478/v10001-010-0023-6 20934956

[B23] Conway A. R. Kane M. J. Bunting M. F. Hambrick D. Z. Wilhelm O. Engle R. W. (2005). Working memory span tasks: a methodological review and user's guide. Psychon. Bull. Rev. 12, 769–786. 10.3758/BF03196772 16523997

[B24] Critchley H. D. Eccles J. Garfinkel S. N. (2013). Interaction between cognition, emotion, and the autonomic nervous system. Handb. Clin. Neurol. 117, 59–77. 10.1016/B978-0-444-53491-0.00006-7 24095116

[B25] Daneman M. Carpenter P. A. (1980). Individual differences in working memory and reading. J. Verb. Learn. Verb. Behav. 19, 450–466. 10.1016/S0022-5371(80)90312-6

[B26] De Vente W. Olff M. Van Amsterdam J. G. C. Kamphuis J. H. Emmelkamp P. M. G. (2003). Physiological differences between burnout patients and healthy controls: blood pressure, heart rate, and cortisol responses. Occup. Environ. Med. 60, i54–i61. 10.1136/oem.60.suppl_1.i54 12782748 PMC1765727

[B27] Dobson C. P. Kim A. Haigney M. (2013). QT Variability Index. Prog. Cardiovasc. Dis. 56, 186–194. 10.1016/j.pcad.2013.07.004 24215750

[B28] Engle R. W. Tuholski S. W. Laughlin J. E. Conway A. R. (1999). Working memory, short-term memory, and general fluid intelligence: a latent-variable approach. J. Exp. Psychol. Gen. 128:309. 10.1037/0096-3445.128.3.309 10513398

[B29] Fishta A. Backé E. M. (2015). Psychosocial stress at work and cardiovascular diseases: an overview of systematic reviews. Int. Arch. Occup. Environ. Health 88, 997–1014. 10.1007/s00420-015-1019-0 25687981 PMC4608992

[B30] Ford D. E. Mead L. A. Chang P. P. Cooper-Patrick L. Wang N. Y. Klag M. J. (1998). Depression is a risk factor for coronary artery disease in men: the precursors study. Arch. Intern. Med. 158, 1422–1426. 10.1001/archinte.158.13.1422 9665350

[B31] Friedman N. P. Miyake A. (2005). Comparison of four scoring methods for the reading span test. Behav. Res. Methods 37, 581–590. 10.3758/BF03192728 16629290

[B32] Gallacher J. Bayer A. Fish M. Pickering J. Pedro S. Dunstan F.. (2009). Does anxiety affect risk of dementia? Findings from the Caerphilly Prospective Study. Psychosom. Med. 71, 659–666. 10.1097/PSY.0b013e3181a6177c 19553290

[B33] Gao S. A. Johansson M. Hammarén A. Nordberg M. Friberg P. (2005). Reproducibility of methods for assessing baroreflex sensitivity and temporal QT variability in end-stage renal disease and healthy subjects. Clin. Auton. Res. 15, 21–28. 10.1007/s10286-005-0224-4 15768198

[B34] Gardener H. Wright C. B. Dong C. Cheung K. DeRosa J. Nannery M.. (2016). Ideal cardiovascular health and cognitive aging in the northern manhattan study. J. Am. Heart Assoc. 5:e002731. 10.1161/JAHA.115.002731 26984255 PMC4943249

[B35] Golkar A. Johansson E. Kasahara M. Osika W. Perski A. Savic I. (2014). The influence of work-related chronic stress on the regulation of emotion and on functional connectivity in the brain. PLoS ONE 9:e104550. 10.1371/journal.pone.0104550 25184294 PMC4153588

[B36] Gottesman R. F. Schneider A. L. Albert M. Alonso A. Bandeen-Roche K. Coker L.. (2014). Midlife hypertension and 20-year cognitive change: the atherosclerosis risk in communities neurocognitive study. JAMA Neurol. 71. 1218–1227. 10.1001/jamaneurol.2014.1646 25090106 PMC4226067

[B37] Grossi G. Perski A. Ekstedt M. Johansson T. Lindström M. Holm K. (2005). The morning salivary cortisol response in burnout. J. Psychosom. Res. 59, 103–111. 10.1016/j.jpsychores.2005.02.009 16186006

[B38] Grossi G. Perski A. Osika W. Savic I. (2015). Stress-related exhaustion disorder–clinical manifestation of burnout? A review of assessment methods, sleep impairments, cognitive disturbances, and neuro-biological and physiological changes in clinical burnout. Scand. J. Psychol. 56, 626–636. 10.1111/sjop.12251 26496458

[B39] Hansen A. L. Johnsen B. H. Thayer J. F. (2003). Vagal influence on working memory and attention. Int. J. Psychophysiol. 48, 263–274. 10.1016/S0167-8760(03)00073-4 12798986

[B40] Hasan M. A. Abbott D. Baumert M. (2012). Relation between beat-to-beat QT interval variability and T-wave amplitude in healthy subjects. Ann. Noninvasive Electrocardiol. 17, 195–203. 10.1111/j.1542-474X.2012.00508.x 22816538 PMC6932369

[B41] Hansen A. L. Johnsen B. H. Sollers III J. J. Stenvik K. Thayer J. F. (2004). Heart rate variability and its relation to prefrontal cognitive function: the effects of training and detraining. Eur. J. Appl. Physiol. 93, 263–272. 10.1007/s00421-004-1208-0 15338220

[B42] Hughes J. W. Stoney C. M. (2000). Depressed mood is related to high-frequency heart rate variability during stressors. Psychosom. Med. 62, 796–803. 10.1097/00006842-200011000-00009 11138999

[B43] Jarczok M. N. Jarczok M. Mauss D. Koenig J. Li J. Herr R. M.. (2013). Autonomic nervous system activity and workplace stressors- a systematic review. Neurosci. Biobehav. Rev. 37, 1810–1823. 10.1016/j.neubiorev.2013.07.004 23891906

[B44] Jolles J. Houx P. J. Van Boxtel M. P. J. Ponds R. W. H. M. (1995). Maastricht Aging Study: Determinants of Cognitive Aging. Maastricht: Neuropsych Publishers.

[B45] Kimhy D. Crowley O. V. McKinley P. S. Burg M. M. Lachman M. E. Tun P. A.. (2013). The association of cardiac vagal control and executive functioning–findings from the MIDUS study. J. Psychiatr. Res. 47, 628–635. 10.1016/j.jpsychires.2013.01.018 23434176 PMC3594003

[B46] Kivimäki M. Nyberg S. T. Batty G. D. Fransson E. I. Heikkilä K. Alfredsson L.. (2012). Job strain as a risk factor for coronary heart disease: a collaborative meta-analysis of individual participant data. Lancet 380, 1491–1497. 10.1016/S0140-6736(12)60994-5 22981903 PMC3486012

[B47] Kivimäki M. Virtanen M. Elovainio M. Kouvonen A. Väänänen A. Vahtera J. (2006). Work stress in the etiology of coronary heart disease—a meta-analysis. Scand. J. Work Environ. Health 32, 431–442. 10.5271/sjweh.1049 17173200

[B48] Kivipelto M. Helkala E. L. Hänninen T. Laakso M. P. Hallikainen M. Alhainen K.. (2001a). Midlife vascular risk factors and late-life mild cognitive impairment a population-based study. Neurology 56, 1683–1689. 10.1212/WNL.56.12.1683 11425934

[B49] Kivipelto M. Helkala E. L. Laakso M. P. Hänninen T. Hallikainen M. Alhainen K.. (2001b). Midlife vascular risk factors and Alzheimer's disease in later life: longitudinal, population based study. BMJ 322, 1447–1451. 10.1136/bmj.322.7300.1447 11408299 PMC32306

[B50] Knowles E. E. Weiser M. David A. S. Glahn D. C. Davidson M. Reichenberg A. (2015). The puzzle of processing speed, memory, and executive function impairments in schizophrenia: fitting the pieces together. Biol. Psychiatry 78, 786–793. 10.1016/j.biopsych.2015.01.018 25863361 PMC4547909

[B51] Koschke M. Boettger M. K. Schulz S. Berger S Terhaar, J. Voss A.. (2009). Autonomy of autonomic dysfunction in major depression. Psychosom. Med. 71, 852–860. 10.1097/PSY.0b013e3181b8bb7a 19779146

[B52] Kristensen T. S. Hannerz H. Høgh A. Borg V. (2005). The copenhagen psychosocial questionnaire-a tool for the assessment and improvement of the psychosocial work environment. Scand. J. Work Environ. Health 31, 438–449. 10.5271/sjweh.948 16425585

[B53] Leineweber C. Baltzer M. Hanson L. L. M. Westerlund H. (2013). Work–family conflict and health in Swedish working women and men: a 2-year prospective analysis (the SLOSH study). Eur. J. Public Health 23, 710–716. 10.1093/eurpub/cks064 22683777 PMC3719472

[B54] Liao D. Cai J. Rosamond W. D. Barnes R. W. Hutchinson R. G. Whitsel E. A.. (1997). Cardiac autonomic function and incident coronary heart disease: a population-based case-cohort study the ARIC study. Am. J. Epidemiol. 145, 696–706. 10.1093/aje/145.8.696 9125996

[B55] Liston C. McEwen B. S. Casey B. J. (2009). Psychosocial stress reversibly disrupts prefrontal processing and attentional control. Proc. Natl. Acad. Sci. U.S.A. 106, 912–917. 10.1073/pnas.0807041106 19139412 PMC2621252

[B56] Luque-Casado A. Perales J. C. Cárdenas D. Sanabria D. (2016). Heart rate variability and cognitive processing: the autonomic response to task demands. Biol. Psychol. 113, 83–90. 10.1016/j.biopsycho.2015.11.013 26638762

[B57] Magrì D. Piccirillo G. Quaglione R. Dell'armi A. Mitra M. Velitti S.. (2012). Effect of acute mental stress on heart rate and QT Variability in Postmyocardial infarction patients. ISRN Cardiol. 2012:912672. 10.5402/2012/912672 22844616 PMC3403409

[B58] Mann S. L. Selby E. A. Bates M. E. Contrada R. J. (2015). Integrating affective and cognitive correlates of heart rate variability: a structural equation modeling approach. Int. J. Psychophysiol. 98, 76–86. 10.1016/j.ijpsycho.2015.07.003 26168884 PMC4980075

[B59] McElree B. (2001). Working memory and focal attention. J. Exp. Psychol. Learn. Mem. Cogn. 27, 813–835. 10.1037/0278-7393.27.3.817 11394682 PMC3077110

[B60] McEwen B. S. (2007). Physiology and neurobiology of stress and adaptation: central role of the brain. Physiol. Rev. 87, 873–904. 10.1152/physrev.00041.2006 17615391

[B61] McEwen B. S. Bowles N. P. Gray J. D. Hill M. N. Hunter R. G. Karatsoreos I. N.. (2015). Mechanisms of stress in the brain. Nat. Neurosci. 18, 1353–1363. 10.1038/nn.4086 26404710 PMC4933289

[B62] Montaquila J. M. Trachik B. J. Bedwell J. S. (2015). Heart rate variability and vagal tone in schizophrenia: a review. J. Psychiatr. Res. 69, 57–66. 10.1016/j.jpsychires.2015.07.025 26343595

[B63] Mueller A. Bonnemeier H. Malberg H. Kurths J. Wessel N. (2014). Age-dependent changes in the manifestations of gender-related differences in the cardiovascular regulation, in 8th Conference of the European Study Group on Cardiovascular Oscillations (ESGCO) (IEEE), 147–148.

[B64] Murrough J. W. Iacoviello B. Neumeister A. Charney D. S. Iosifescu D. V. (2011). Cognitive dysfunction in depression: neurocircuitry and new therapeutic strategies. Neurobiol. Learn. Mem. 96, 553–563. 10.1016/j.nlm.2011.06.006 21704176

[B65] Olsen L. R. Jensen D. V. Noerholm V. Martiny K. Bech P. (2003). The internal and external validity of the Major Depression Inventory in measuring severity of depressive states. Psychol. Med. 33, 351–356. 10.1017/S0033291702006724 12622314

[B66] Olsson E. M. Roth W. T. Melin L. (2010). Psychophysiological characteristics of women suffering from stress-related fatigue. Stress Health 26, 113–126. 10.1002/smi.1271

[B67] Peters A. McEwen B. S. (2015). Stress habituation, body shape and cardiovascular mortality. Neurosci. Biobehav. Rev. 56, 139–150. 10.1016/j.neubiorev.2015.07.001 26148986

[B68] Petkus A. J. Reynolds C. A. Wetherell J. L. Kremen W. S. Pedersen N. L. Gatz M. (2015). Anxiety is associated with increased risk of dementia in older Swedish twins. Alzheimers Dement. 12, 399–406. 10.1016/j.jalz.2015.09.008 26549599 PMC4841698

[B69] Piccirillo G. Cacciafesta M. Lionetti M. Nocco M. Di Giuseppe V. Moisè A.. (2001). Influence of age, the autonomic nervous system and anxiety on QT-interval variability. Clin. Sci. (Lond). 101, 429–438. 10.1042/cs1010429 11566081

[B70] Piccirillo G. Rossi P. Mitra M. Quaglione R. Dell'Armi A. Barba D.. (2013). Indexes of temporal myocardial repolarization dispersion and sudden cardiac death in heart failure: any difference? Ann. Noninvasive Electrocardiol. 18, 130–139. 10.1111/anec.12005 23530483 PMC6932543

[B71] Richard Jennings J. Allen B. Gianaros P. J. Thayer J. F. Manuck S. B. (2015). Focusing neurovisceral integration: cognition, heart rate variability, and cerebral blood flow. Psychophysiology 52, 214–224. 10.1111/psyp.12319 25160649 PMC4387874

[B72] Sandström A. Rhodin I. N. Lundberg M. Olsson T. Nyberg L. (2005). Impaired cognitive performance in patients with chronic burnout syndrome. Biol. Psychol. 69, 271–279. 10.1016/j.biopsycho.2004.08.003 15925030

[B73] Seldenrijk A. Vogelzangs N. Batelaan N. M. Wieman I. van Schaik D. J. Penninx B. J. (2015). Depression, anxiety and 6-year risk of cardiovascular disease. J. Psychosom. Res. 78, 123–129. 10.1016/j.jpsychores.2014.10.007 25454680

[B74] Shipley B. A. Der G. Taylor M. D. Deary I. J. (2008). Cognition and mortality from the major causes of death: the Health and Lifestyle Survey. J. Psychosom. Res. 65, 143–152. 10.1016/j.jpsychores.2008.02.017 18655859

[B75] Sindi S. Hakansson K. Hagman G. Kulmala J. Soininen H. Kareholt I. (2014). Mid-life work-related stress increases dementia risk in late-life: the CAIDE 30-year study. Alzheimers Dement. 10, 746 10.1016/j.jalz.2014.05.1408 27059705

[B76] Stenfors C. U. Marklund P. Hanson L. L. M. Theorell T. Nilsson L. G. (2013). Subjective cognitive complaints and the role of executive cognitive functioning in the working population: a case-control study. PLoS ONE 8:e83351. 10.1371/journal.pone.0083351 24386185 PMC3873297

[B77] Steptoe A. Kivimäki M. (2012). Stress and cardiovascular disease. Nat. Rev. Cardiol. 9, 360–370. 10.1038/nrcardio.2012.45 22473079

[B78] Stroop J. (1935). Studies of interference in serial verbal reactions. J. Exp. Psychol. 18, 643–661. 10.1037/h0054651

[B79] Takada M. Ebara T. Kamijima M. (2010). Heart rate variability assessment in Japanese workers recovered from depressive disorders resulting from job stress: measurements in the workplace. Int. Arch. Occup. Environ. Health 83, 521–529. 10.1007/s00420-009-0499-1 20012751

[B80] Thayer J. F. Hansen A. L. Saus-Rose E. Johnsen B. H. (2009). Heart rate variability, prefrontal neural function, and cognitive performance: the neurovisceral integration perspective on self-regulation, adaptation, and health. Ann. Behav. Med. 37, 141–153. 10.1007/s12160-009-9101-z 19424767

[B81] Thayer J. F. Yamamoto S. S. Brosschot J. F. (2010). The relationship of autonomic imbalance, heart rate variability and cardiovascular disease risk factors. Int. J. Cardiol. 141, 122–131. 10.1016/j.ijcard.2009.09.543 19910061

[B82] Theorell T. Jood K. Järvholm L. S. Vingård E. Perk J. Östergren P. O. Hall C. (2016). A systematic review of studies in the contributions of the work environment to ischaemic heart disease development. Eur. J. Public Health 26, 470–477. 10.1093/eurpub/ckw025 27032996 PMC4884330

[B83] Toker S. Shirom A. Shapira I. Berliner S. Melamed S. (2005). The association between burnout, depression, anxiety, and inflammation biomarkers: C-reactive protein and fibrinogen in men and women. J. Occup. Health Psychol. 10:344. 10.1037/1076-8998.10.4.344 16248685

[B84] Tombaugh T. N. (2003). Trail making test A and B: normative data stratified by age and education. Arch. Clin. Neuropsychol. 19, 203–214. 10.1016/S0887-6177(03)00039-8 15010086

[B85] Tsuji H. Venditti F. J. Jr. Manders E. S. Evans J. C. Larson M. G. Feldman C. L.. (1994). Reduced heart rate variability and mortality risk in an elderly cohort. The Framingham Heart Study. Circulation 90, 878–883. 10.1161/01.CIR.90.2.878 8044959

[B86] Ursin H. Eriksen H. R. (2010). Cognitive activation theory of stress (CATS). Neurosci. Biobehav. Rev. 34, 877–881. 10.1016/j.neubiorev.2009.03.001 20359586

[B87] Vandeput S. Verheyden B. Aubert A. E. Van Huffel S. (2008). Nonlinear heart rate variability in a healthy population: influence of age. Comput. Cardiol. 35, 53–56. 10.1109/cic.2008.4748975

[B88] Voss A. Schroeder R. Heitmann A. Peters A. Perz S. (2015). Short-term heart rate variability—influence of gender and age in healthy subjects. PLoS ONE 10:e0118308. 10.1371/journal.pone.0118308 25822720 PMC4378923

[B89] Wang H. X. Wahlberg M. Karp A. Winblad B. Fratiglioni L. (2012). Psychosocial stress at work is associated with increased dementia risk in late life. Alzheimers Dement. 8, 114–120. 10.1016/j.jalz.2011.03.001 22404853

[B90] Watkins L. L. Koch G. G. Sherwood A. Blumenthal J. A. Davidson J. R. O'Connor C.. (2013). Association of anxiety and depression with all-cause mortality in individuals with coronary heart disease. J. Am. Heart Assoc. 2:e000068. 10.1161/JAHA.112.000068 23537805 PMC3647264

[B91] Wulsin L. R. Horn P. S. Perry J. L. Massaro J. M. D'Agostino R. B. (2015). Autonomic imbalance as a predictor of metabolic risks, cardiovascular disease, diabetes, and mortality. J. Clin. Endocrinol. Metab. 100, 2443–2448. 10.1210/jc.2015-1748 26047073

[B92] Yeragani V. K. Tancer M. Uhde T. (2003). Heart rate and QT interval variability: abnormal alpha-2 adrenergic function in patients with panic disorder. Psychiatry Res. 121, 185–196. 10.1016/S0165-1781(03)00235-X 14656453

